# Majocchi’s granuloma on the forearm caused by *Trichophyton tonsurans* in an immunocompetent patient

**DOI:** 10.1186/s12941-020-00382-y

**Published:** 2020-09-02

**Authors:** Yun-yan Zheng, Yue Li, Ming-yan Chen, Qian-yun Mei, Ru-zhi Zhang

**Affiliations:** Department of Dermatology, The Third Affiliated Hospital of Soochow University, Changzhou, 213003 Jiangsu China

**Keywords:** Majocchi’s granuloma, Trichophyton tonsurans, Itraconazole, Forearm

## Abstract

Majocchi's granuloma is an uncommon fungal infection of the dermis and subcutaneous tissue. The most frequently identified cause of Majocchi’s granuloma is anthropophilic *Trichophyton rubrum*, and it is most commonly located on the anterior aspect of the lower limbs in women. Here, we report a case of Majocchi’s granuloma on the forearm, a site that is rarely involved, in a 62-year-old woman who had been bitten by a dog. Histological examination revealed a dense dermal infiltrate composed of lymphoplasmacytic cells and neutrophils, with hyphae in the dermis. The presence of the fungus, *Trichophyton tonsurans*, was confirmed by mycological examination and molecular methods. Therefore, histological and mycological examination confirmed the diagnosis of Majocchi’s granuloma. The patient was treated with local moxibustion and itraconazole, 200 mg/day, for 60 days, which facilitated a complete resolution of the lesions.

## Background

Majocchi's granuloma is an uncommon presentation of skin infection caused by invasion of fungus, which is divided into two types according to the patient's health condition and clinical manifestations. The first type is perifolliculitis that occurs mainly in healthy individuals and in the lower limbs, it is usually caused by penetrating trauma, clinical manifestations usually appear as perifollicular papules. The second type is subcutaneous nodules that usually happens in immunosuppressed hosts and in the upper limbs, clinical manifestations characterized by groups of nodules [1]. *Trichophyton rubrum* is the most common cause of Majocchi’s granuloma, followed by *Trichophyton mentagrophytes*, *Trichophyton violaceum*, and *Trichophyton tonsurans* [2]. In this case report, an immunocompetent patient developed nodules after trauma and we confirmed that the pathogen was *Trichophyton tonsurans*. All these characteristics are different from common Majocchi’s granuloma. The patient was cured by oral itraconazole after 2 months of treatment.

## Case report

A 62-year-old female patient presented to our hospital with a complaint of a lump on her left forearm for 5 months, and the condition had been aggravated for the last month. The lump was not painful or itchy. The patient had her left forearm bitten by a dog 5 months prior. At that time, she was treated at a pet hospital and injected with a rabies vaccine. The wound healed very slowly but failed to heal completely, leaving a small red nodules. In the month prior, the red nodules had increased significantly in size without pain or itching, and there was a small amount of pus after squeezing. She self treated with an oral cephalosporin for one week. No topical treatments, including topical steroids, were used. Her condition did not improve, so she came to our hospital for treatment. She had no history of any other disease.

**Fig. 1:**
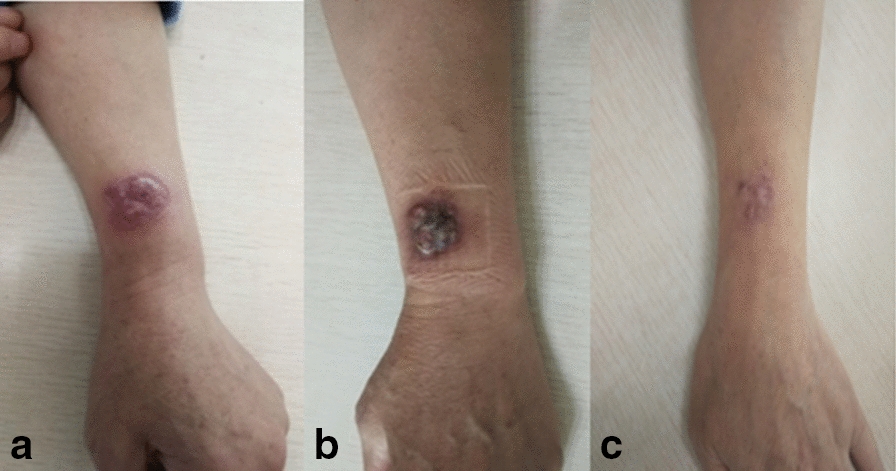
Clinical manifestation on the patient’s forearm before and after treatment. **a** Before treatment—Clinical photograph showing a 3 × 3.5 cm lump on her left forearm, with a clear boundary, a few scales and scabs on the surface. **b** After 2 weeks of treatment with oral itraconazole and moxibustion, the lump was swollen and exuded pus. **c** After 2 months of treatment, Majocchi’s granuloma was completely absorbed and changed to scars

Physical examination revealed a 3 × 3.5 cm sized lump on her left forearm, with a clear boundary, a few scales and scabs on the surface, and no tenderness (Fig. 1a). Her trunk and limbs were free of tinea corporis, tinea pedis and onychomycosis.

**Fig. 2:**
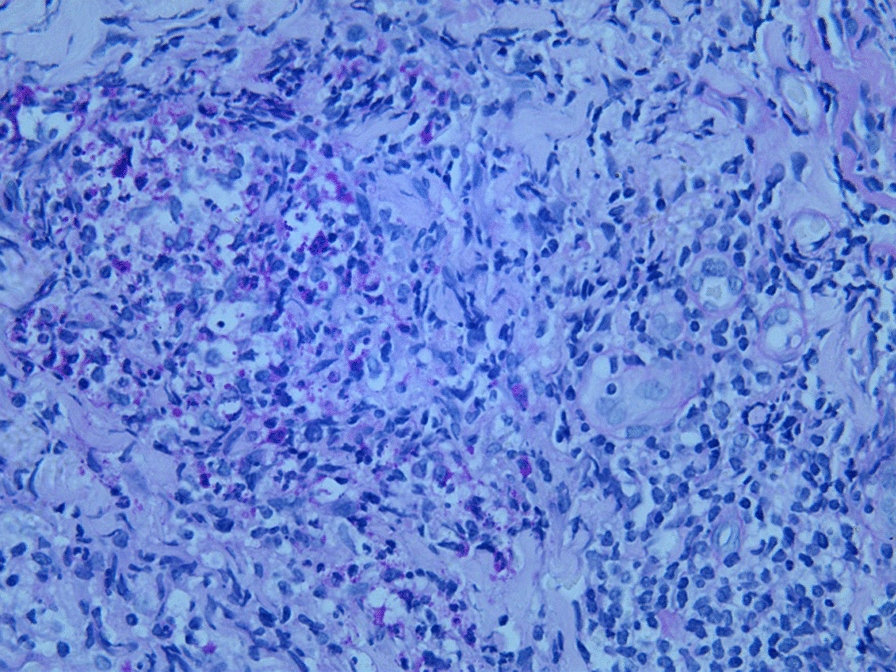
PAS staining. Hyphae were found in the dermis (PAS staining, × 400)

Laboratory investigations showed normal liver, kidney, immune function test results and T lymphocyte subsets were normal. The results of human immunodeficiency virus (HIV) were negative. Fungal microscopy was negative. PAS staining was positive for hyphae (Fig. 2).

**Fig. 3:**
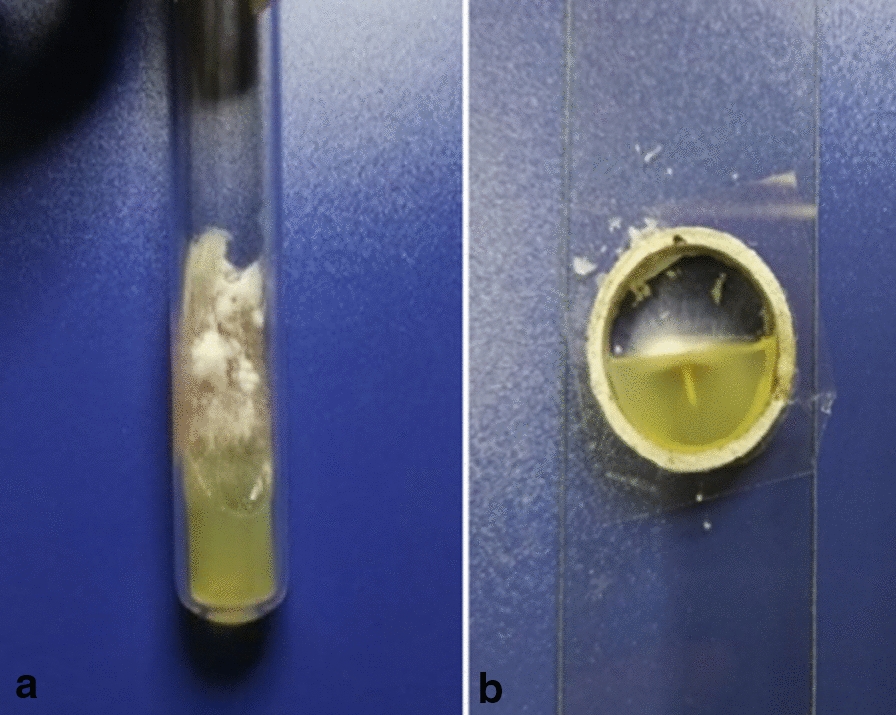
Fungal cultures. Fungal cultures showing white velvety colonies (**a**) and white colonies in small cultures (**b**)

**Fig. 4:**
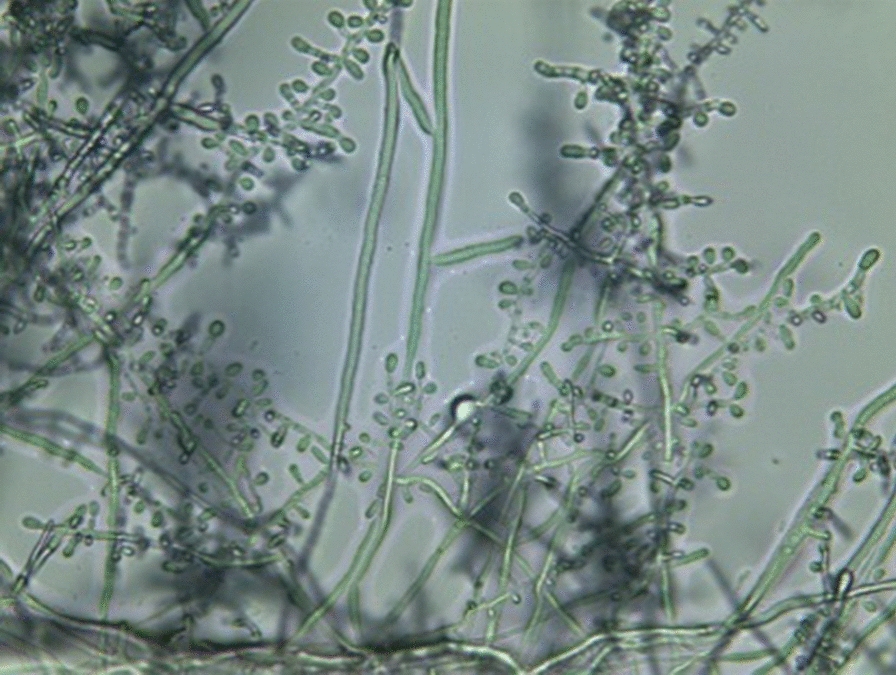
Fungal small cultures. Fungal small cultures revealed a large number of small conidia of different shapes with the ends of individual small conidia enlarged, like balloons

A fungal culture was performed in a 27 °C incubator and showed the fungi growing slowly with a variable texture. The surfaces of colonies were velvety (Fig. 3a). White colonies could be seen in small cultures (Fig. 3b). A large number of small conidia of different shapes were seen in small cultures, and the ends of individual small conidia enlarged, like balloons (Fig. 4). The results showed only the growth of *Trichophyton tonsurans*.

**Fig. 5:**
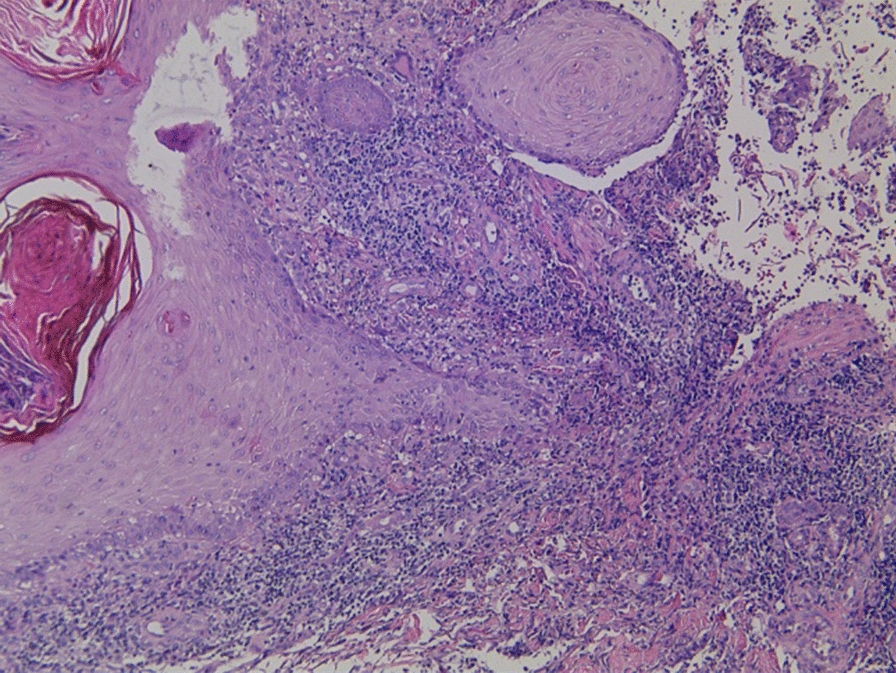
Histological examination. Histological examination showing epithelioma-like hyperplasia of the spinous layer

Histological examination showed excessive keratinization of the epidermis and epithelioma-like hyperplasia of the spinous layer (Fig. 5). Dense lymphocytes and neutrophils infiltrated the superficial dermis. The above characteristics are consistent with infectious granulomatous changes. Molecular identification showed that the fungus was 99.7% similar to AB094063.1 *Trichophyton tonsurans* in GenBank, and was eventually diagnosed as *Trichophyton tonsurans* through ITS molecular diagnosis (ITS1, ITS4).

The patient was given itraconazole 100 mg twice daily. At the same time, the patient was given moxibustion treatment, which used a burning moxa to stimulate the lumps twice a day for 20 min each time. At the beginning of the moxibustion, the lumps were swollen and exuded pus (Fig. 1b), and there was pain, but it was tolerable. After 2 weeks of treatment, the lumps began to be absorbed. After 40 days, the lumps were further absorbed and there was no exudation of pus on their surfaces. The treatment lasted for a total duration of 2 months until the lesions of Majocchi’s granuloma were completely absorbed and changed to scars (Fig. 1c). There was not any recurrence of Majocchi’s granuloma after the treatment at a 3-months follow-up.

## Discussion

Majocchi’s granuloma is an uncommon fungal infection that is generally seen among patients presenting with skin tinea and immunocompromised diseases. The long-term use of steroids, chemotherapy or antineoplastic therapy or other immunosuppressive conditions may have a higher risk of developing Majocchi’s granuloma [2]. However, immunodeficiency is not a necessary condition to diagnose Majocchi’s granuloma since several immunocompetent patients with Majocchi's granuloma have been reported [3–5].

The source of Majocchi’s granuloma can be a prior dermatophyte infection, exposure to infected or asymptomatic animals or humans, and local or general immunosuppressive conditions. The main cause of Majocchi’s granuloma is *Trichophyton rubrum*, but in this case, we showed that *Trichophyton tonsurans* caused the Majocchi's granuloma through histological and mycological examination.

Several cases of Majocchi's granuloma caused by *Trichophyton tonsurans* have been reported, but most of those patients also suffered from diseases that cause immunodeficiencies, such as AIDS [6], organ transplants [7], etc. Nodular granulomatosis in the arm of an immunocompetent host caused by *Trichophyton tonsurans* has rarely been reported. However, to further confirm the diagnosis of Majocchi’s granuloma, a series of auxiliary examinations should be performed, such as tissue pathology, PAS staining, GMS staining and fungal culture.

Dermatophytes degrade the keratin in nonliving keratinized tissues to survive. However, in the case of Majocchi’s granuloma, the mechanism whereby the fungus survived in the dermis and subcutaneous tissue is unclear. We speculate that it is likely related to an impaired skin barrier. In this case, the patient was free of other diseases that may cause immunocompromised conditions, but had her left forearm bitten by a dog before. Therefore, we speculated that the Majocchi’s granuloma in this patient may occur because of damage to the integrity of the epidermal barrier and follicular disruption after being bitten by a dog. Then, fungi, along with keratin and necrotic materials, entered the dermis and caused an inflammatory response during infection.

There are no consensus guidelines for the treatment of Majocchi’s granuloma. Some dermatologists recommend oral antifungal agents such as terbinafine or itraconazole and the duration of therapy should be at least 4–8 weeks and should be continued until all lesions are resolved [3, 8]. This case is being reported as it demonstrates the atypical location of Majocchi’s granuloma in the forearm of an immunocompetent host, who had been successfully treated with local moxibustion and oral itraconazole.

## Data Availability

All data are fully available without restriction.
